# A Circular Polarizer with Beamforming Feature Based on Frequency Selective Surfaces

**DOI:** 10.1038/srep41505

**Published:** 2017-01-27

**Authors:** Jia Yuan Yin, Xiang Wan, Jian Ren, Tie Jun Cui

**Affiliations:** 1 State Key Laboratory of Millimeter Waves, Southeast University, Nanjing 210096, China; 2 Synergetic Innovation Center of Wireless Communication Technology, Southeast University, Nanjing, 210096, China; 3 State Key Laboratory of Millimeter Waves and Department of Electronic Engineering, City University of Hong Kong, Kowloon, Hong Kong SAR, China; 4 Cooperative Innovation Center of Terahertz Science, Chengdu 611731, China

## Abstract

We propose a circular polarizer with beamforming features based on frequency selective surface (FSS), in which a modified anchor-shaped unit cell is used to reach the circular polarizer function. The beamforming characteristic is realized by a particular design of the unit-phase distribution, which is obtained by varying the scale of the unit cell. Instead of using plane waves, a horn antenna is designed to feed the phase-variant FSS. The proposed two-layer FSS is fabricated and measured to verify the design. The measured results show that the proposed structure can convert the linearly polarized waves to circularly polarized waves. Compared with the feeding horn antenna, the transmitted beam of the FSS-added horn is 14.43° broader in one direction, while 3.77° narrower in the orthogonal direction. To our best knowledge, this is the first time to realize circular polarizer with beamforming as the extra function based on FSS, which is promising in satellite and communication systems for potential applications due to its simple design and good performance.

Circularly polarized (CP) waves play an important role in satellite and communication systems owing to their special characteristics. When being used in the aerospace communication system and remote sensing devices, CP waves can reduce the signal loss efficiently and eliminate the polarization influence caused by Faraday rotation effect in ionosphere. In electronic countermeasures, CP waves are used to detect and disturb any other polarization-mode waves except for the reversed CP waves. More importantly, the CP antenna on high-speed objects or rolling objects can receive radio signals under arbitrary position. Hence the methods to produce CP waves have intrigued great interests over the years. Various kinds of methods have been proposed to convert linearly polarized (LP) waves to CP waves. Such as employing chiral^1^,^2^,^3^,^4^ or photonic metamaterials^5^,^6^,^7^, waveguide^8^,^9^,^10^,^11^, different kinds of slots^12^,^13^, meander line^14^,^15^,^16^, grating structures^17^,^18^,^19^, metasurfaces^20^,^21^,^22^, and so on. Among all the conversion ways, frequency selective surfaces (FSS) that provide different characteristics for horizontal and vertical components of incident LP waves are widely used due to their simple designs and good performance^23^,^24^,^25^,^26^,^27^,^28^. By adjusting the unit-cell structure, FSS can be equivalent to a capacitance for one component of incident wave, while be equivalent to an inductance for the other component. Then the phase difference between the two components can be easily designed as ±90°. If the magnitude of the two components is equal at the same time, the conversion from LP waves to CP waves is accomplished. Based on these analyses, ref. 24 proposed a wideband multilayer circular polarizer based on bisected split-ring FSS. The wideband feature is at the expense of a bulky structure of four layers. Experimental results showed that axial ratios lower than 3 dB are obtained over the frequency range from 25.5 to 36.5 GHz.

However, different circumstances need different radiation patterns. FSS that can provide different radiation patterns have been desiderated^29^,^30^,^31^,^32^. If FSS with the CP characteristic has effects on radiation pattern simultaneously (e.g. beamforming), then it can be used in many different practical applications. Beamforming is such a kind of technology that realize particular radiation pattern by calculating the weight of every unit cells of an array. There are mainly two approaches to realize beamforming. One is optimizing the arrangement mode of the unit cells, and the other is optimizing the phase distribution of the unit cells. Taking the features of FSS into consideration, the second method is more appropriate in the design of beamforming FSS.

In this paper, a circular polarizer with beamforming feature is proposed using FSS structure. The modified anchor-shaped unit cell is used to realize the conversion from LP to CP waves, taking the advantage of its different characteristics for different polarizations. The phase of each unit cell can be changed by varying the scale of the unit cell. Thus on the basis of the antenna array theory, the beamforming characteristic can be realized by a particular design of the phase distribution. In order to make the proposed structure more practical, a horn antenna is designed to feed FSS, instead of using plane waves. The proposed FSS is fabricated in a circular area with a 60 mm radius. Both simulated and measured results show that the proposed structure can convert LP to CP waves efficiently. Meanwhile, compared with the radiation pattern of the feeding horn antenna, the transmission beam is 14.43° broader in one direction, while in the orthogonal direction, the transmission beam is 3.77° narrower after the FSS is added.

## Results

**Figure 1:**
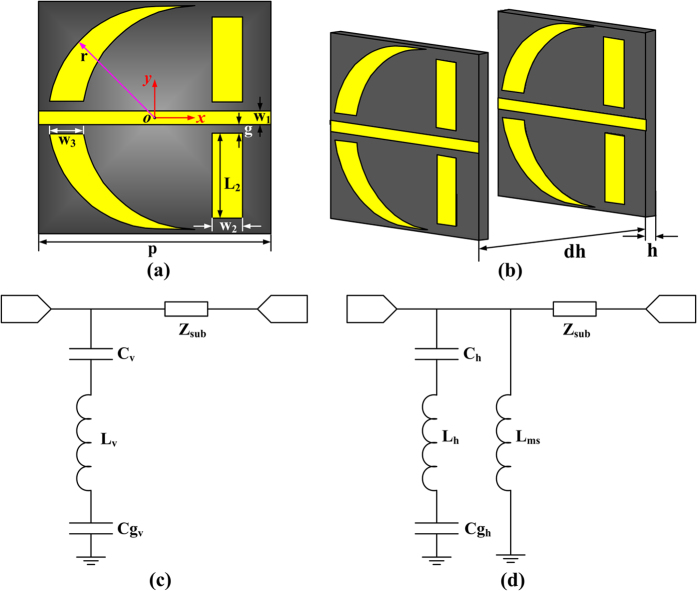
Schematic of the modified anchor-shaped unit cell, in which the yellow part is metal (modeled as copper) and the grey part is the substrate. (**a**) Planar layout of a unit cell. (**b**) Unit cell of the two-layer FSS. (**c**) Equivalent circuit models of the single-layer FSS to *y*-polarized incident waves. (**d**) Equivalent circuit models of the single-layer FSS to *x*-polarized incident waves.

The unit cell of the proposed FSS is based on an anchor-shaped structure^33^ with slots, as shown in Fig. 1(a). The slots are introduced to realize different characteristics for horizontal and vertical components of the incident LP waves. The whole structure is composed of two layers separated by a distance of 12 *mm* in order to obtain the expected phases, which is determined through the optimization. The proposed FSS is fabricated on a 1 *mm*-thick substrate with a relative permittivity of 2.65 and loss tangent of 0.003. The thickness of metallic layer is 0.018 *mm*. In this particular design, the periodicity p of unit cell is 7 *mm*. Other geometry parameters are as follows: r = 6.6 *mm*, w1 = 1.2 *mm*, w2 = 1.8 *mm*, w3 = 2 *mm*, and L2 = 5 *mm*. It is worth noting that unit cells at different layers have the same orientation, and are aligned with respect to their centers rigorously.

According to the coordinate system in Fig. 1, we consider an incident LP wave, whose electric field vector 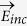 is oriented at *θ* = 45° to the *y*-axis. Then the incident LP waves can be decomposed into two orthogonal LP components of equal magnitude, along the *x*- and *y*-axis, respectively. Based on the definition of CP waves, when the magnitudes of the two components are equal and the phase difference reaches 90°, the incident LP wave is converted to the CP wave successfully.

To make the conversion principle more clearly, an equivalent circuit model is given in Fig. 1(c and d), which are similar to what ref. 24 shows. Different responses are corresponding to different components. Figure 1(c) shows the equivalent circuit model for the y-polarization, and Fig. 1(d) gives the equivalent circuit model for the x-polarization. Here, C_gv_ and C_gh_ represent the effects of slots on the vertical polarization (*y*-polarization) and horizontal polarization (*x*-polarization), respectively. The inductance introduced by the metal strip in the middle of the structure is represented by L_ms_ in this figure, while C_v_, C_h_, L_v_ and L_h_ are the equivalent capacitances and inductances of other parts of the metal structure. In the same way, the circuit model of the two-layer circular polarizer is generated by cascading two stages connected with transmission lines, where the air space between the two layers are taken into consideration in the transmission lines. Then by adjusting the values of the capacitances and inductances, which can be implemented by varying the dimensions of the metal structure, the condition to realize the CP wave mentioned above can be satisfied. It is worth noting that the circuit model only provides a general way to explain how the phase shifts are provided. The values of equivalent inductances and capacitances are changed as the scaling factor varies, while the circuit model remains unchanged. There are some limitations on the circuit model indeed. The phase difference between the two components cannot always keep 90° when the scaling factor changes, which will have a slight impact on the circular polarization effect. The details of the changes on phase difference will be discussed in the next part.

**Figure 2:**
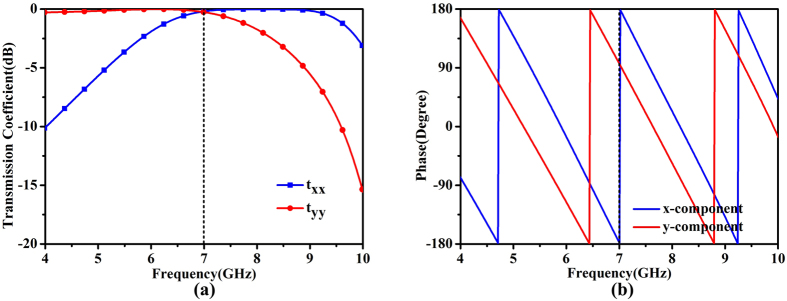
Simulated results of the unit cell. (**a**) Amplitudes of transmission coefficients of the unit cell. (**b**) Phase difference between the two orthogonal components.

For confirmation, the amplitudes of transmission coefficients of the initial unit cell are simulated and presented in Fig. 2(a), while Fig. 2(b) given the phase difference between the two components. The electric field vector of the incident plane wave is oriented at *θ* = 45° to the *y*-axis and propagating along the –*z* direction. Here, *t*_*xx*_ represents the transmission coefficient of the *x*-polarized wave, and *t*_*yy*_ signifies the transmission coefficient of the *y*-polarized waves. It is obviously that the transmitted magnitudes of the two components are substantially equal and their phase difference reaches 90° exactly at 7 GHz. Thus the circular polarizer is realized by the two-layer FSS.

**Figure 3:**
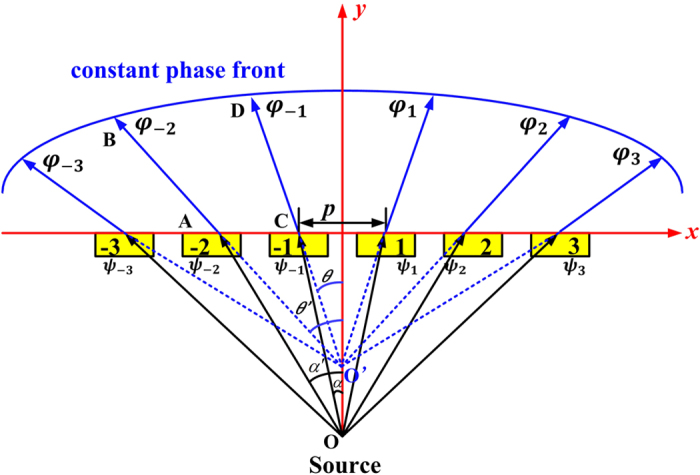
Schematic of the phase differences caused by different positions of the unit cells in broadening the beam.

Next, the beamforming property must be taken into account in this particular design. The beamforming technology is such a kind of technology that can realize the specific radiation pattern by calculating the weight of every unit cell of an array. There are mainly two approaches to realize the beamforming feature. One is to optimize the arrangement mode of the unit cells, and the other is to optimize the phase distribution of the unit cells. In this particular design, the latter one is used. The main step of this method can be described as follows. Firstly, an expected radiation pattern needs to be decided. Then the phase difference from the point source to the expected phase front should be calculated. To ensure the phase differences among the entire light path keep the same, the phase compensation of each unit cell is determined automatically. The final step is to organize the array. One of the most important applications of the beamforming is to broaden the transmission beam. Figure 3 illustrates the principle of beam broadening in the case of point-source excitation. Because the point source is more closely to the practical situation, there are phase differences caused by the point source. When the electromagnetic waves radiate from a point source (Point O), there will be phase difference between the different positions of the plane with a certain distance from the source. Consider the simplified model that the different positions along a line (*x*-axis). We use Point A to represent patch numbered −2, and Point C to represent patch numbered −1. Because of the different paths between OA and OC, the electromagnetic waves reach Point A and Point C own different transmission phase. This is part of the phase difference between the two unit cells and we use Δ*ψ* to represent this kind of difference. The feeding phase differences are expressed by *ψ*_*−3*_, *ψ*_*−2*_, *ψ*_*−1*_, *ψ*_*0*_, *ψ*_*1*_, *ψ*_*2*_, *ψ*_*3*_, corresponding to the unit cells numbered from −3 to 3. Taking the unit cells numbered by −1 and −2 as an example, the feeding phase difference between the two unit cells can be described as:


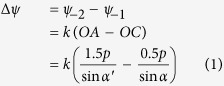


where *k* is the wavenumber, *p* is the period of the unit cell. Afterwards, an expected phase front should be determined. According to the expected phase front, the different paths for electromagnetic waves transmitted through Point A and Point C are decided by Line AB and CD. This difference also contributes to the phase difference between the two unit cells. When the electromagnetic waves transmit through FSS, the phase differences caused by the different paths from FSS to the expected constant phase front can be expressed as:


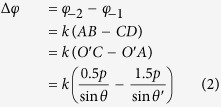


where O′ is the virtual image of the source of the broaden beam. *φ*_*−3*_, *φ*_*−2*_, *φ*_*−1*_, *φ*_*0*_, *φ*_*1*_, *φ*_*2*_, *φ*_*3*_ are the phase changes from the FSS to the constant phase front. Finally, the total phase difference between unit cells is a combination of these two differences. As a consequence, the phase difference between unit cells numbered by −2 and −1 is


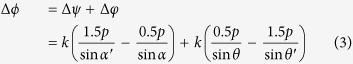


Since the distance from the source to FSS and the beam broadening degree are already setting, then *α, α*′, *θ, θ*′ are determined. Finally the expected phase difference to realize beam broadening can be calculated. The principle is similar when the beam is required to be narrowed. The only difference is that the virtual image of the source is on the other side of the real source.

**Figure 4:**
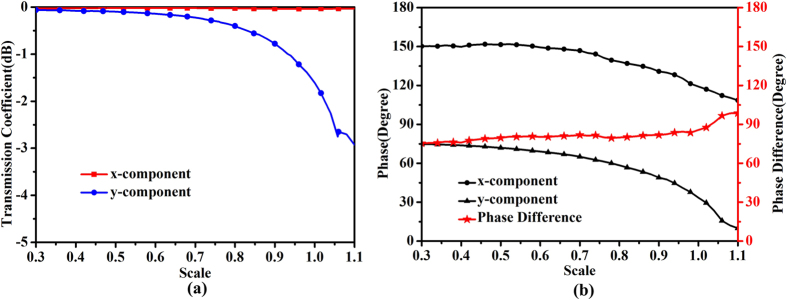
(**a**) Transmission coefficients and (**b**) Phases of the two orthogonal components under different unit scales, as well as the difference between the two components at 7 GHz.

Based on the above theoretical analysis, the transmission phase of each unit cell should be confirmed. Figure 4 shows the transmission coefficient and phase trend when changing the scale of the metal structure at 7 GHz. It is observed that the magnitudes of two components keep relatively stable except for the reduction of *y*-component under the high scaling factor. But the magnitude of *y*-component can be still larger than −3 dB, indicating the availability of the large-scale unit cell. The phase difference between the two components is also given in this figure. We notice that a phase difference of 90° ± 10° is obtained around the frequency of 7 GHz. The phase difference between the two components cannot always keep 90°when the scaling factor changes. The reason of this fact may result from the unscaled thickness of FSS. That is to say, a unique substrate is used for all unit cells, and the scaling is only applied for the grid feature. Fortunately, the highly scaled cells, located on the edge of FSS, have relatively less contribution to generate the CP waves. That is to say, the effect of the central cells (with less scaling factor) is strong enough so as to dominate the edge cells and still create the CP waves. The unit cell in this paper can be regarded as a modification of the unit cell in ref. 33, the result of which shows a good stability of the anchor-shaped unit cell. But the unfixed phase difference may also give rise to the instability of the proposed structure. This is mainly because the same size of the unit cells is used in the whole structure in ref. 33.

**Figure 5:**
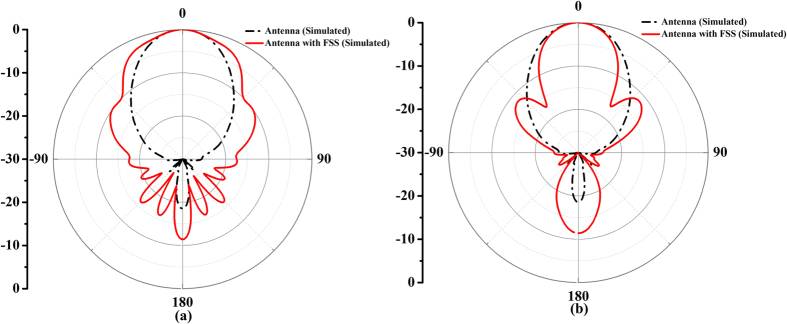
Comparison between the simulated and measured normalized transmission patterns at 7 GHz of the horn antenna and the horn antenna with FSS added. (**a**) Beam-broadened direction. (**b**) Beam-narrowed direction.

Once the unit cells are scaled down, their characteristics will be affected. The stability becomes worse consequently. According to the certain rules mentioned above, the transmission phase of each unit cell can be confirmed, from which the corresponding scale of the unit cell is determined. As an application of the proposed design, we require that the beam is broadened in one direction while is narrowed in the other direction. Then the whole phase changing curve should be divided equally into two parts. One is used to broaden the transmission beam; the other is used to narrow the transmission beam. The final scaling factors of the unit cells numbered from −3 to 3 are 1.1, 1.02, 0.96, 0.96, 1.02, and 1.1. For the other direction along the *y*-axis, the final scaling factors of the unit cells from the middle to both sides are set as 0.3, 0.62, and 0.96. After the scale of each unit cell is decided, the whole FSS can be modeled for simulation. Although the FSS structure is determined, there is still a certain distance before the design can be used in the practical applications. Taking the reality into consideration, a relevant horn antenna is designed, and thus the proposed circular polarizer can be put in front of the horn antenna directly. If a circular polarized beam with the specific radiation pattern is needed, then the horn antenna with FSS can be taken out to realize the expected beam. It is worth noting that the above analysis is based on the point source, but the horn antenna is not an ideal point source. Most LP horn antennas have different phase centers at different planes. Geometrical optics, being analyzed above, is a general explanation of the operating principle. The equivalent phase center can be considered at the average value of the two phase centers. So the phase center of the horn antenna was confirmed through the simulation by CST Microwave Studio. The simulated results at 7 GHz of the final structure composed of two-layer FSS and a horn antenna are presented in Fig. 5(a and b), comparing with the horn antenna, demonstrating the abilities to transform LP to CP and independently beamforming feature. The black lines signify the simulated results while the red lines show the measured results. It is apparently that the patterns of the two orthogonal directions owns opposite deformation. One is broadening and the other is narrowing.

**Figure 6:**
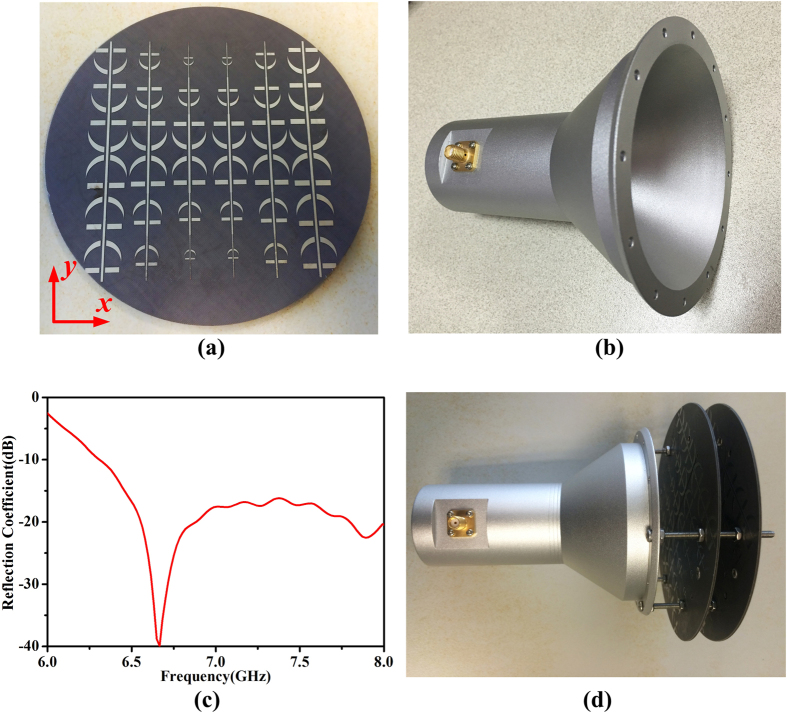
(**a**) Photograph of one layer of the proposed two-layer FSS. (**b**) Photograph of the horn antenna. (**c**) Measured reflection coefficient of the designed horn antenna. (**d**) Final assembled sample of the proposed structure and the experiment is implemented through the nearfield measurement system.

To confirm the proposed design, we fabricated and measured a circular-polarizer FSS with the radius of 60 *mm*. The photograph of one of the fabricated layers is shown in Fig. 6(a). According to the coordinate system given in the figure, the final beam-broadened direction is along the *x*-axis while the beam-narrowed direction is along the *y*-axis. It is observed that the upper half is the mirror-image of the lower half of FSS. The mirror-image of the proposed structure is just made for the symmetry of the metal pattern. If the proposed structure is mirrored in the two rows at center, the performance will not change. Although the unit cells are designed based on the assumption of local periodicity, we have found that when the unit cell is rotated around its center, the transmission feature keeps unchanged. So the orientation of each unit cell is not a key part to the performance of the whole structure. Figure 6(b) is the designed horn antenna for verification of the principle analyzed above. The measured reflection coefficient of the horn antenna is given in Fig. 6(c). With reference to the figure, it is obviously that the reflection coefficient of the horn antenna is less than −10 dB in a wide range of frequency, indicating the good impedance match in the design of horn antenna. And it can be speculated that the SWR of the horn antenna is less than 1.5 in the operating frequency band. The final assembled sample is given by Fig. 6(d), in which the proposed two-layer FSS is fixed in the front of the antenna by a distance of 20 *mm* with screws, and the middle metal strip is oriented 45° to the axis of the horn antenna. The distance between the two layers is set up as of 12 *mm* in the experiment. The distance between the horn and the first FSS and that between the two layers is only a test value. Other values can be used here as long as it is considered when calculating the phase distribution.

**Figure 7:**
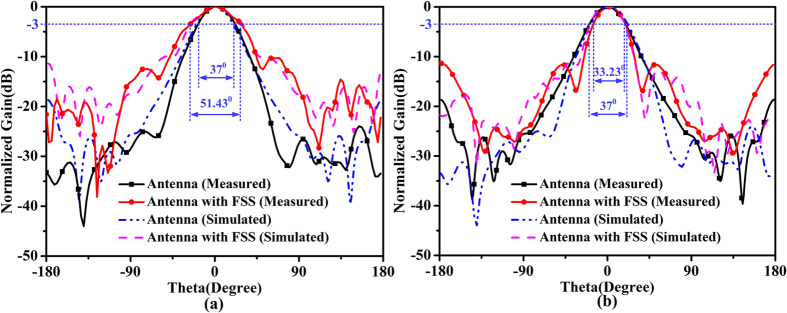
Comparison of measured two-dimensional transmission patterns of the horn antenna with and without FSS at 7 GHz. (**a**) Beam-broadened direction. (**b**) Beam-narrowed direction.

For comparisons, the measured results of the horn only are also presented. We note that all measured results agree well with numerical simulations. To have a clear recognition of the beam changes, Fig. 7(a and b) give the comparative simulated and measured transmission patterns of the horn antenna with and without FSS on two planes at 7 GHz, respectively. The solid lines show the measured results, while the dashed lines give the simulated ones. In the measured results, the black lines are the radiation patterns of the horn antenna, and the red lines denote the FSS-added horn antenna. From the comparison, it is apparent that the beam is broadened in the designed direction while is narrowed in the orthogonal direction at the designed frequency. The 3 dB bandwidth of the horn antenna is 37°, while that of the FSS-added horn antenna in beam-broadened direction and beam-narrowed direction achieve 51.43° and 33.23°, respectively. That is to say the broadening angle is about 14.43°, and the narrowing angle is about 3.77°. Here the 3 dB bandwidth is used to represent the width of the beam. We observe that there is a slight difference between the simulated and measured results, especially in the backward radiation. The main cause of the difference maybe comes from the assembly error in the experiment. The measurement system and the support at the bottom of the horn antenna both have effect on the radiation patterns, while the simulation through the CST software is always calculated under ideal condition.

**Figure 8:**
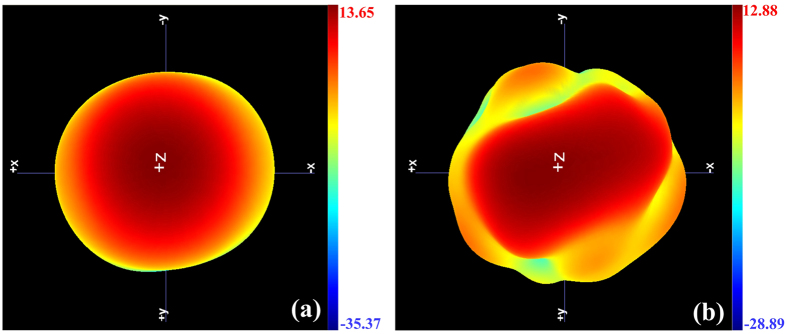
Measured three-dimensional transmission patterns at 7 GHz. (**a**) Horn antenna only. (**b**) Horn antenna with the proposed FSS.

Meanwhile, it is crucial to note that as for a common prescribed radiation pattern, when broadening the beam width in one plane, the beam in orthogonal plane is narrowing accordingly. But the situation of this particular design is that the incident electromagnetic wave is decomposed into two orthogonal LP waves when transmitting through the proposed structure. And the two components are controlled by different mechanisms. So each component has its own radiation pattern individually. Figure 8 provides the measured three-dimensional far-field pattern at 7 GHz for intuitive understanding of the beam changes. We can clearly see the changes of the radiation patterns. The circular shaped pattern presented by horn antenna becomes elliptical look, for the different deformation in the two directions. Table 1 is another comparison between simulated and measured results. From the table we see that the measured gain of the FSS-added horn antenna is 12.88 dBi, while the horn antenna itself owns a gain of 13.65 dBi. Moreover, the efficiency of the FSS-added horn antenna reaches 94.89% in experiments. From the two results, we note that the gain and efficiency of the proposed structure are lower than those of horn antenna only. This is mainly caused by the shape of the expected radiation pattern. Generally speaking, the gain is enhanced when the energy is focused to one point. While the energy is scattered, the gain is reduced inevitably. In this particular design, the energy is not focused to one point, resulting in the reduction of gain and efficiency. What’s more, the gain loss of the FSS-added horn antenna is also caused by the dielectric loss of FSS. The measured results are competent to indicate the good performance of the proposed FSS.

**Figure 9:**
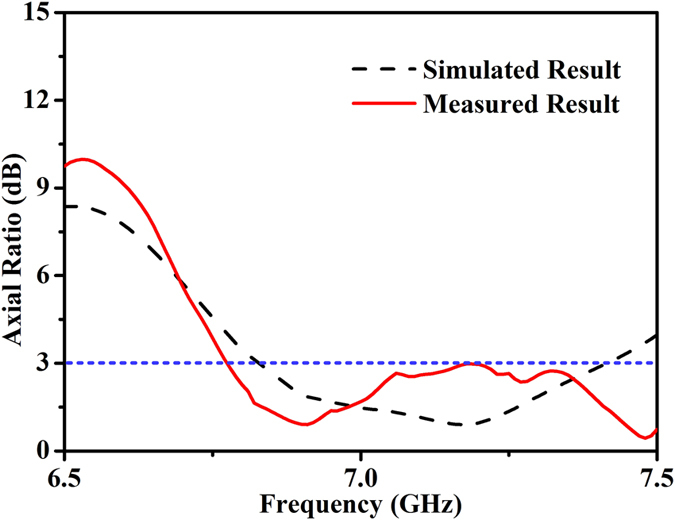
Measured axial ratio of the FSS-added horn antenna at 7 GHz.

To see the effect of polarization conversion, the axial ratio in the main lobe direction of the proposed design is also simulated and measured, as demonstrated in Fig. 9. From the reference in this figure, the measured axial ratio in solid red line is less than 3 dB at the designed frequency and has a certain bandwidth above 6.8 GHz, confirming the previous design that the FSS acts as a circular polarizer. However, it is noteworthy that the infinitely periodic structure was considered in the simulation, while a prototype with finite dimension was measured in the experiment. This contributes to the imperfect axial ratio compared with the simulated results. In addition, the fabrication tolerances, non-optimal distance between two layers, angular misalignment of the electric field vector of the incident wave with the axis, misalignment between the two layers, and the border effects can give rise to the larger axial ratios in the experiment. It is known that, the expected radiation pattern, in which the circularly-polarized beam width is broadened in one direction and narrowed in the orthogonal direction, can also be realized by putting the normal beamforming FSS in front of the circular horn antenna fed with two orthogonal ports with equal amplitude and 90-degree phase difference. But the key point becomes the shape of radiation pattern instead of the beam feature. The proposed FSS structure is a circular polarizer primarily, and then is used to change the shape of the radiation pattern.

**Figure 10:**
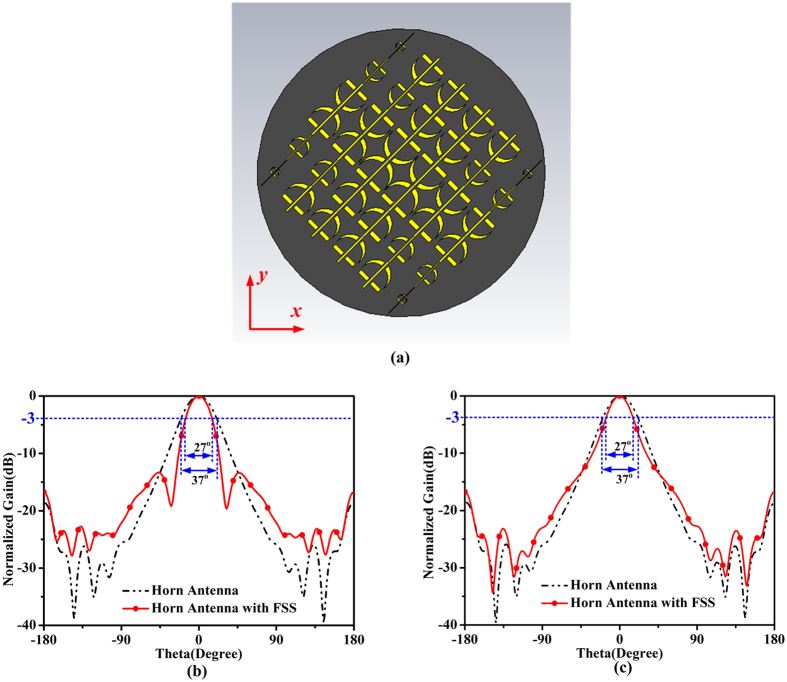
(**a**) Schematic of one of the two layers FSSs with gain enhancement feature. (**b**) and (**c**) are the comparison between radiation patterns of the horn antenna with and without FSS in two directions at 7 GHz.

In fact, there are many possibilities for beamforming using the proposed theory. Here we consider the other circumstance to enhance the gain of the transmission beam. The radiation patterns in the both directions are narrowed so that the total gain of this particular design is improved, conforming to the fact that gain enhancement can be realized if the energy is converged to one point. Using the proposed method, we design the FSS structure for this purpose, as illustrated in Fig. 10(a), in which the scales of unit cells in each line of the two directions will diminish from the middle to edge. Two layers FSSs are also needed in this particular design. Figure 10(b and c) give the comparison of transmission normalized radiation patterns of the horn antenna with (red lines) and without (black lines) the new designed FSS at the same operating frequency 7 GHz. It is obviously that the beam widths in both directions are narrowed after the FSS structure shown in Fig. 10(a) is put in front of the horn antenna. The beam-widths of the FSS-added horn antenna in both directions become 27°, differing from the 37° of the horn antenna. The simulated gain of the original horn antenna is 13.6 dBi, while with the help of FSS, the gain achieves 15.1 dBi. This design can also verify the fact that the two components are controlled individually.

## Discussion

In this work, a circular polarizer with beamforming feature based on FSS has been proposed. The conversion from LP to CP waves owes to the modified anchor-shaped unit cell, taking the advantages of its different characteristics for different polarized waves. The beamforming characteristic is achieved by a particular design of the unit phase distribution. Different transmission phases are obtained by varying the scale of the unit cell. A horn antenna is designed to feed the FSS structure, instead of the plane wave. The experimental results show that the proposed structure can successfully convert the LP waves to CP waves, with the axial ratio less than 3 dB at the main lobe direction. Compared with the radiation patterns of the horn antenna, the transmission beam of the FSS-added antenna is 14.43° broader in one direction, while is 3.77° narrower in the other direction. To the best knowledge of the authors, this is the first time to realize the circular polarizer with beamforming as extra function by the use of FSS. The proposed circular polarizer with beamforming character is of great values as functional radome in satellite and communication systems due to its simple design and good performance.

## Methods

Numerical simulations are performed by the commercial software, CST Microwave Studio. The experimental structure is fabricated using a 1-*mm* thin dielectric film with dielectric constant 3 and tangent loss 0.03, respectively. The thickness of metal (copper, a kind of lossy metal, the conductivity of which is 5.8 × 10^7^ S/m) film is 0.018 *mm*. We use Agilent Vector Network Analyzer to measure the *S* parameters (i.e., the reflection coefficients *S*_*11*_) of the fabricated sample. The transmission patterns of the horn antenna with the proposed FSS are measured by the nearfield measurement system Satimo Starlab.

## Additional Information

**How to cite this article**: Yin, J. Y. *et al*. A Circular Polarizer with Beamforming Feature Based on Frequency Selective Surfaces. *Sci. Rep.* **7**, 41505; doi: 10.1038/srep41505 (2017).

**Publisher's note:** Springer Nature remains neutral with regard to jurisdictional claims in published maps and institutional affiliations.

## Figures and Tables

**Table 1:** Simulated and measured total gain and efficiency of the horn antenna with and without the proposed fss.

	Gain (dBi)		Efficiency	
	Simulated	Measured	Simulated	Measured
Horn	13.6	13.65	99.7%	96.52%
Horn with FSS	12.6	12.88	98.67%	94.89%
